# 1,2-Hydrogenation and Transhydrogenation Catalyzed by 3-Ketosteroid Δ^1^-Dehydrogenase from *Sterolibacterium denitrificans*—Kinetics, Isotope Labelling and QM:MM Modelling Studies

**DOI:** 10.3390/ijms232314660

**Published:** 2022-11-24

**Authors:** Agnieszka M. Wojtkiewicz, Michał Glanowski, Piotr Waligórski, Tomasz Janeczko, Maciej Szaleniec

**Affiliations:** 1 Jerzy Haber Institute of Catalysis and Surface Chemistry, Polish Academy of Sciences, Niezapominajek 8, 30-239 Krakow, Poland; 2 The Franciszek Górski Institute of Plant Physiology, Polish Academy of Sciences, Niezapominajek 8, 30-239 Krakow, Poland; 3 Department of Food Chemistry and Biocatalysis, Wrocław University of Environmental and Life Sciences, Norwida 25, 50-375 Wrocław, Poland

**Keywords:** 3-ketosteroid dehydrogenase, KstD, flavin enzyme, transhydrogenation, hydrogenation

## Abstract

Bacteria and fungi that are able to metabolize steroids express 3-ketosteroid-Δ^1^-dehydrogenases (KstDs). KstDs such as AcmB form *Sterolibacterium denitrificans* Chol-1 catalyze the enantioselective 1α,2β-dehydrogenation of steroids to their desaturated analogues, e.g., the formation of 1,4-androstadiene-3,17-dione (ADD) from 4-androsten-3,17-dione (AD). The reaction catalyzed by KstD can be reversed if the appropriate electron donor, such as benzyl viologen radical cation, is present. Furthermore, KstDs can also catalyze transhydrogenation, which is the transfer of H atoms between 3-ketosteroids and 1-dehydrosteroids. In this paper, we showed that AcmB exhibits lower pH optima for hydrogenation and dehydrogenation by 3.5–4 pH units than those observed for KstD from *Nocardia corallina.* We confirmed the enantiospecificity of 1α,2β-hydrogenation and 1α,2β-transhydrogenation catalyzed by AcmB and showed that, under acidic pH conditions, deuterons are introduced not only at 2β but also at the 1α position. We observed a higher degree of H/D exchange at Y363, which activates the C2-H bond, compared to that at FAD, which is responsible for redox at the C1 position. Furthermore, for the first time, we observed the introduction of the third deuteron into the steroid core. This effect was explained through a combination of LC-MS experiments and QM:MM modelling, and we attribute it to a decrease in the enantioselectivity of C2-H activation upon the deuteration of the 2β position. The increase in the activation barrier resulting from isotopic substitution increases the chance of the formation of d^3^-substituted 3-ketosteroids. Finally, we demonstrate a method for the synthesis of 3-ketosteroids chirally deuterated at 1α,2β positions, obtaining 1α,2β-d^2^-4-androsten-3,17-dione with a 51% yield (8.61 mg).

## 1. Introduction

3-Ketosteroid-Δ^1^-dehydrogenases (KstD) represent a large group of FAD-dependent enzymes produced by bacteria and fungi that catalyze the regio- and enantioselective 1,2-dehydrogenation of 3-ketosteroids to their 1-dehydro derivatives [1,2]. An example of such a process is the dehydrogenation of 4-androsten-3,17-dione (AD) to 1,4-androstadiene-3,17-dione (ADD) [3,4]. The dehydrogenation of AD and other 3-ketosteroids, such as 9α-hydroxy-4-androstene-3,17-dione [5,6], catalyzed by KstDs, is a critical reaction in the environmental cholesterol degradation pathway, initiating a ring A opening pathway and a central degradation pathway of the sterane ring system [7,8,9]. It also has industrial applications in the production of API and synthones for further modification [1]. Moreover, KstDs are essential for the pathogenicity of *Mycobacterium tuberculosis*, which causes tuberculosis [7].

The catalytic mechanism of the oxidative 1,2-dehydrogenation reaction was mainly studied based on the available structures of KstD1 from *Rhodococcus erythropolis* [8] (PDB: 4C3Y) and the recently resolved AcmB (anaerobic cholesterol metabolism enzyme B, PDB: 7P18) from *Sterolibacterium denitrificans* Chol-1 [10,11]. The analysis of KstDs amino acid sequences revealed that AcmB represents a large sub-group of KstDs containing a membrane-associated domain (MAD), previously referred to as a “loop” [10,12,13]. Based on the structural studies supported by site-directed mutagenesis, kinetic isotope effect (KIE) studies and QM:MM MD modelling, the reaction mechanism of 1,2-dehydrogenation catalyzed by AcmB was described in detail. 1,2-Dehydrogenation catalyzed by KstDs proceeds according to ping-pong bi-bi kinetic mechanisms, which can be divided into oxidative half-reaction (OHR), during which the steroid is oxidized and the FAD is reduced, and reductive half-reaction (RHR), during which the other substrate (a natural or artificial electron acceptor such as, e.g., 2,6-dichloroindophenol) is reduced and the FADH^−^ is reoxidized [9]. The RHR proceeds according to the previously suggested Ec2b two-step mechanism [1,9,14]. The reaction starts when deprotonated tyrosine (Y363), acting as a base, enantioselectively abstracts 2β protons from the steroid and leads to the formation of the carbanion intermediate, stabilized by the H-bond with tyrosine residues present at the active site (Tyr536, Tyr118 and Y363). In the next step, the hydride is transferred from the 1α position to N5 of the FAD. After the release of the 1-dehydro-steroid in RHR, the re-oxidant binds to the active site and reoxidizes FADH^−^ to FAD [9].

The reversibility of 1,2-dehydrogenation catalyzed by KstDs was briefly studied in the early 1990s in the last century based on KstD from *Nocardia corallina* [10,15]. The transhydrogenation of progesterone to 1,4-androstdien-3,17-dione proceeded at the same rate as the dehydrogenation of 3-keto-4-ene-steroid. The pH optimum for transhydrogenation (8.2) was in the middle of the pH optima for hydrogenation (6.6) and dehydrogenation (10.0) [10]. As expected, the mechanism also proceeded according to the ping-pong bi-bi mechanism, with 1-dehydrosteroid acting as the FADH^−^ reoxidation agent [10].

**Figure 1:**
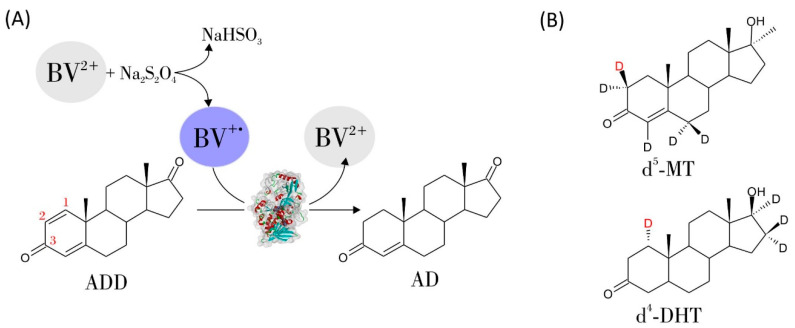
(**A**) The scheme of the 1,2-hydrogenation of ADD to AD catalyzed by AcmB from *S. denitrificans* with benzyl viologen (BV^+^) as an electron donor, generated in situ by reducing BV^2+^ with sodium dithionite (Na_2_S_2_O_4_). (**B**) Deuterium-labelled substrates used in transhydrogenation experiments: 2,2,4,6,6-d^5^-17-methyl-testosterone (d^5^-MT) and 1,16,16,17-d^4^-dihydrotestosterone (d^4^-DHT). Deuterons H2β at C2 of d^5^-MT and H1α at C1 of d^4^-DHT are marked in red.

In this work, we continued the work of Itagaki et al. from 30 years ago. We aimed to further elucidate the 1,2-hydrogenation mechanism and enantioselectivity of substrate activation. To achieve this, we conducted experiments with AD and ADD as a transhydrogenation couple and steroids deuterated at the C1 or C2 position (Figure 1B), and QM:MM modelling. We also present a synthesis method for isotopically labelled steroids via 1,2-hydrogenation, where deuterons from D_2_O can be enantioselectively introduced at the C1 and C2 positions.

## 2. Results and Discussion

### 2.1. 1,2-Hydrogenation

We studied the kinetics of the 1,2-hydrogenation of ADD to AD through a UV–Vis assay following a change in the absorbance of BV^+^ to BV^2+^. In the reaction, the BV^+·^ generated in situ reduced FAD in AcmB to FADH^−^, which, in turn, reduced ADD to AD (Figure 1A).

#### 2.1.1. pH Dependency

**Figure 2:**
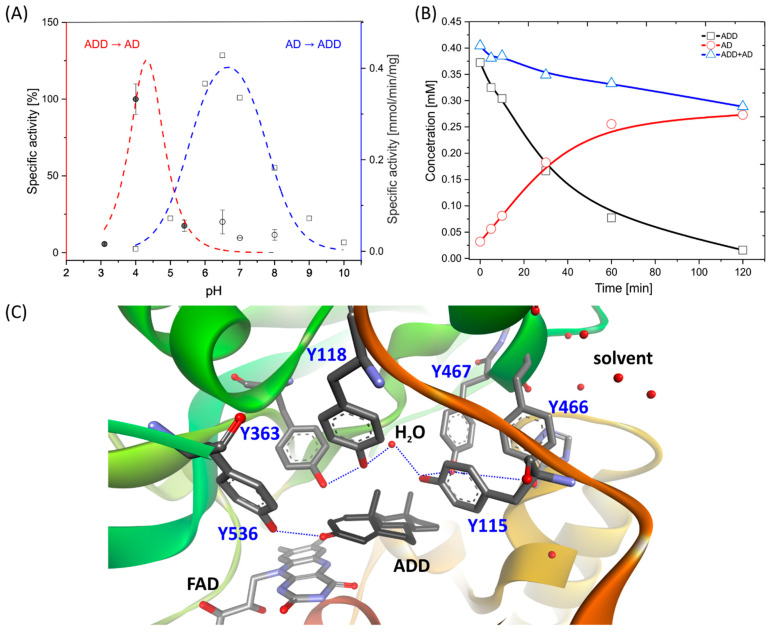
1,2-Hydrogenation catalyzed by AcmB from *S. denitrificans* Chol-1 with ADD as a substrate and BV^+^ as an electron donor in the presence of dithionite. (**A**) The pH optimum of the reaction obtained through a spectrophotometric assay, red line and grey circles—activities measured in 50 mM of citrate buffer, white circles—activities measured in 50 mM of phosphate buffer compared to the pH optimum for 1,2-dehydrogenation catalyzed by AcmB with DCPIP as an electron acceptor ([11], changed), blue line, white squares. (**B**) The progress of 1,2-hydrogenation at pH 6.5 in a fed-batch reactor monitored by HPLC: squares ADD, circles AD, triangles sum of ADD and AD. (**C**) Active site of AcmB with ADD bound at the active site with a chain of tyrosines forming catalytic machinery and the proton relay system.

First, we investigated the pH dependency of 1,2-hydrogenation, and the obtained kinetic data were fitted with a bell-shaped model. The maximum was observed at a pH of 4.3 (Figure 2A), with a pK_a1_ value of 6.06 and a pK_a2_ of 2.6. The drop in the activity for a pH pK_a2_ below of 2.6 is most probably associated with enzyme deactivation due to denaturation. This hypothesis was confirmed by the meagre reduction of ADD at pH 4.0, where only a 1% conversion to AD after 20 h was observed. Furthermore, our previous stability studies showed that AcmB is stable only in the range of pH 6.0–9.0, with a gradually decreasing enzyme activity below 6.0 [11]. Due to this, the further hydrogenation reactor tests were conducted at a pH of 6.5, i.e., the lowest pH at which the enzyme was stable.

On the other hand, we could associate the pK_a1_ value (6.06) with the protonation of Y363 (Figure 2C) or, more broadly, with the protonation of the whole proton relay system. The protonated Y363 was required to deliver a proton at C2 to the carbanion intermediate, which is formed after the transfer of hydride from FADH^−^ to ∆^1^-steroid. This result coincided with the pH optimum of 6.7 and pK_a1_ of 5.5, observed for the 1,2-dehydrogenation of steroids with 2,6-dichloroindophenol (DCPIP) as the electron acceptor [11], and could be interpreted as the deprotonation of the Y363 or the whole proton relay system (see Supplemental Figure S1). Assuming this hypothesis is correct, one would expect that in the pH range of 5.5–6.1, a shift in the proton content of the proton relay system would occur, favoring the dehydrogenation or hydrogenation process. This issue, unfortunately, is far more complicated. Recently, we demonstrated with pre-steady-state kinetics of the half-reaction cycles (i.e., reductive (RHR) or oxidative (OHR) half-reaction cycles), that the pH dependency of dehydrogenation was flat between 7.6 and 10.0, with a small pH optimum at pH 9.0, while it was the oxidation of the reduced enzyme with DCPIP which proceeded preferentially under slightly acidic conditions [9]. Therefore, it seems that the Y363/proton relay system can act as a deprotonated base in a very wide pH range, and the sharp rise in the activity at pK_a1_ of 5.5 is associated with the DCPIP reduction/FADH^−^ oxidation process. It is worth noting that the difference between the pH optima for hydrogenation and dehydrogenation of approximately 2 pH units for AcmB from *S. denitrificans* (4.3 and 6.5) is much narrower than in the case of KstD from *N. corallina* (6.6 and 10.0) [10]. Furthermore, it seems that the active site of AcmB shifts the pK_a_ of the residues responsible for the protonation or deprotonation of the steroids by approximately 3 pH units.

#### 2.1.2. Synthesis of 1α,2β-Deuterated-AD

There are several methods of the catalytic hydrogenation of α,β-unsaturated ketones, which have recently been reviewed by Meemken and Baiker [12] and Hagiwara [13]. The hydrogenation of the C=C bond is predominantly conducted by H_2_ over supported Pd catalysts, while asymmetry is induced by a chiral modifier such as (*S*)-proline, cinchona alkaloids, or amines, although a few examples of other transition metal catalysts, such as Cu/C or Cu/SiO_2_, were also reported [13,16]. The other approach is the application of homogenous transition metal catalysts usually containing iridium, rhodium or iron ions coordinated by bulky ligands that enforce an enantiomeric interaction with the substrate. Such methods were used for the selective hydrogenation of C=C in ring A of 3-ketosteroids [17,18,19,20]. These methods often require harsh conditions (such as an elevated temperature or high H_2_ pressure) or expensive homogenous transition metal catalysts that must be meticulously removed from the product before their pharmaceutical application.

In contrast, biological methods operate under far more benign conditions and do not require expensive ingredients. On the other hand, chemical methods are universal and are not limited by the substate specificity of the enzymatic catalysts. To date, several biotransformation methods have been reported that use fungal species to reduce the substrate. However, in whole-cell biotransformations, one has limited control over the enzymes that are induced by the substrate. This frequently results in the unselective reduction of both the C=C and C=O bonds in steroids [21,22]. Therefore, applying isolated enzymes (or recombinant whole-cell systems) with recognized regio- and stereoselectivity seems to be the best approach [10,23].

The 1,2-hydrogenation process catalyzed by AcmB turned out to proceed significantly slower compared to 1,2-dehydrogenation. The apparent specific enzyme activities of hydrogenation and dehydrogenation at their respective pH optima, i.e., at pH 4.3 for the hydrogenation of 0.4 mM ADD with 0.1 mM BV^+·^ compared to the dehydrogenation at pH 6.5 of 0.4 mM AD with 0.1 mM DCPIP, were 2.43 μM min^−1^ mg^−1^ and 111.6 μM min^−1^ mg^−1^, respectively. This indicates that, in these conditions, hydrogenation proceeds 46-fold slower than dehydrogenation. When the experiment was conducted at the same pH of 6.5, the difference was even higher, as the 1,2-hydrogenation process (0.44 μM min^−1^ mg^−1^) proceeded 254-fold slower than 1,2-dehydrogenation. When both processes were compared at pH 6.5 in a reactor test, the average activity after 60 min of 1,2-dehydrogenation was 30 times higher than that of 1,2-hydrogenation (Table S1). Interestingly, the pH optimum of ADD hydrogenation by KstD from *N. corallina* was 6.6 [10].

Next, we conducted 1,2-hydrogenation in D_2_O in a fed-batch reactor with AcmB as a catalyst and ADD as a substrate at a pD of 6.9. After 1 h of the reaction, the mixture was supplemented with an additional 25 mM of Na-dithionite to shift the equilibrium toward AD (by keeping a high concertation of BV^+·^), thus reproducing the procedure used by Itagaki et al. [10]. The average specific activity during the first hour of the reaction was estimated as 489 µM mg^−1^ h^−1^ (Figure 2B). Although ADD was quickly converted to AD, some of the steroids were trapped within the matrix of the over-reduced BV, which is not soluble in water. This effect was even more visible in the experiment carried out for 45 h, where out of 17 mg of the ADD, a 91% apparent conversion was reached. However, only 8.67 mg of the d^2^-AD could be isolated from the solution and brownish BV precipitate, which gave an overall yield of the reaction of 51%. The over-reduction of a violet BV^+·^ to brown BV was also observed in previous studies, where BV formed a permanent film or precipitate, e.g., when electrochemically reduced in aqueous solutions [24]. It is therefore recommended that one adjusts the Na-dithionite concentration carefully, keeping the BV^+·^ state, while avoiding over-reduction with an excessively high concentration of dithionite to the BV neutral form.

Based on the LC-MS analysis, a major product of the reaction was d^2^-4-androsten-3,17-dione (d^2^-AD), with a molecular mass of 288 *m*/*z* (quasi-molecular ion [M + H]^+^ = 289 *m*/*z*) (Supplementary Figure S1). This result was consistent with the accepted molecular mechanism of the reaction catalyzed by KstD, as one deuteron was transferred from FADD^−^ (which was reduced by BV^+·^ in D_2_O) and the other was transferred from Y363 deuterated by D_2_O. The purified d^2^-AD product was characterized by NMR in CDCl_3_ or DMSO-d^6^, confirming the presence of deuterons at the 1α and 2β axial positions (Figure S2–S15c).

Due to the reduction in the Overhauser effect of the signals from carbon atoms substituted with deuterium [25] in the ^13^C NMR spectrum of the isolated product, a decrease in the intensity of the two signals was observed. Comparing this spectrum (^13^C NMR for 1α,2β-deuterated-AD) with the ^13^C NMR spectrum of ADD (Supplementary Materials), it was unambiguously established that deuterium substitution occurred at the carbon atoms C-1 and C-2. As a result of the analysis of the ^1^H NMR spectrum, it was observed that the signals from the two protons were extinguished. Based on the correlation spectra (COSY, HSQC and HMBC) and the comparison of the chemical shifts and signal multiplicity (^1^H NMR for 1α,2β-deuterated-AD) with the ^1^H NMR spectrum of ADD, we identified that the signals from the protons located in positions 1α and 2β disappeared (Supplementary Materials). On this basis, the structure of the obtained product was defined as 1α,2β-deuterated-4-androstene-3,17-dione.

**Figure 3:**
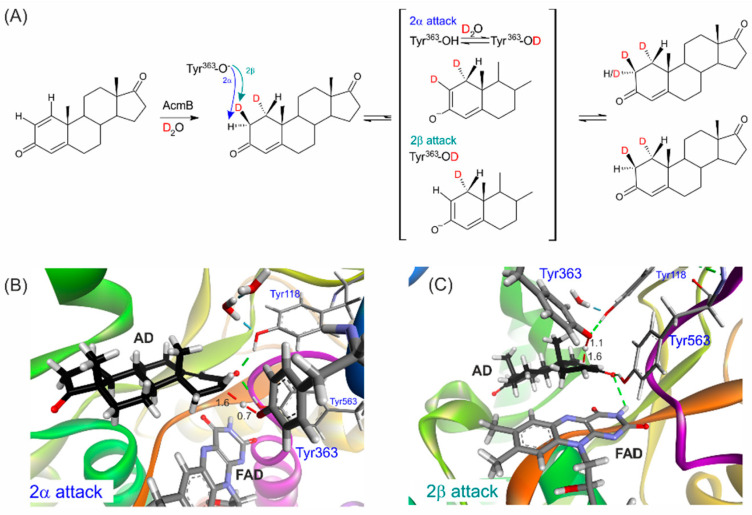
(**A**) 1,2-Hydrogenation of ADD to d^2^-AD in D_2_O followed by isotope exchange at the C2 yielding d^3^-AD. (**B**) Transition state for C2-2αH abstraction by Y363. (**C**) Transition state for the abstraction of 2βH-C2 by Y363. The numbers indicate distances in Å, and green dashed lines indicate H-bond interactions.

Interestingly, at the retention time of AD, we also observed a 290 *m*/*z* signal with an intensity significantly higher (by *ca.* 20%) than that originating from the [M + 1 + H]^+^ isotopic signal of d^2^-AD (i.e., 21% of 289 *m*/*z*, see Supplementary Materials, Figure S1 and Table S2). The 290 *m*/*z* signal slowly increased its abundance with the reaction time, reaching a 33% higher value after 195 h of the reaction, corresponding to 9% of the content of d^3^-AD in the total AD produced in the hydrogenation process. Unfortunately, the amount of this isotopologue was too low to be identified by NMR after its isolation. As a result, we could not experimentally determine the position of the third deuteron introduced in the hydrogenation process. However, the only deuterium source in the hydrogenation was D_2_O. Therefore, the third deuteron had to be introduced by the enzyme into the already chirally deuterated d^2^-AD at either the C1 or C2 atom. As the FAD was kept in a reduced state by the surplus of BV^+·^, the reaction could not reverse toward ADD, and no exchange at 1β-C1 was possible (assuming that FAD could transfer the hydride to the equatorial position). Only the first step of dehydrogenation, i.e., proton abstraction at the C2 atom, could still proceed. When the substrate was bound to the active site, it could interact with tyrosine 363 ion, and the exchange at 2α position could occur (Figure 3). Therefore, we expected the formed product characterized by the 290 *m*/*z* signal to be 1α,2α,2β-d^3^-AD.

The KstDs preferentially abstract the proton from the axial 2β position [2,9]. However, the dehydrogenation of steroids substituted at 2β (e.g., by OH group—see M. Hayano et al. [17]) can still proceed, although at a significantly reduced pace. In our experiment, we applied a substitution of protium with deuterium at the 2β position, which did not exclude its activation but could increase the barrier associated with that process. Therefore, we expected a decrease in C-H activation enantioselectivity upon the deuteration of the 2β axial position.

To test this hypothesis, we conducted QM:MM calculations using a previously developed methodology [14]. We calculated the transition state for C2-2αH or C2-2βD activation in 1α,2β-d^2^-AD and compared it with the same situation for AD without deuterons. The energy barriers, corrected with zero-point energies ∆(E + ZPE), were 9.94 kcal/mol for the activation of the 2βH-C2 bond (Figure 3B) and 14.05 kcal/mol for 2αH-C2 (Figure 3C, Table 1). The energy difference of 4.1 kcal/mol resulted in a 920-fold faster abstraction of 2βH compared to 2αH. As expected, introducing deuterons into AD resulted in primary and secondary kinetic isotope effects. As a result, the barrier for the activation of C2-2βD in d^2^-AD was 10.85 kcal/mol and that for C2-2αH was 14.19 kcal/mol. In effect, a slight reduction in the energy difference between these barriers to 3.35 kcal/mol translated to a 260-fold faster abstraction of 2βD compared to the abstraction of 2αH from the C2 atom. It should be underlined that the isotope exchange rate of Y363 also somewhat diminished the efficiency of the deuteron introduction in place of protium (Figure 3A). However, as we demonstrated in the transhydrogenation experiment conducted in H_2_O (see below), this process proceeded quite readily.

To further confirm our hypothesis, we conducted an isotope exchange experiment using the BV^+·^ reduced enzyme in D_2_O and with 0.01–0.1 mM AD. In such a setup, the 1,2-dehydrogenation reaction could not proceed (due to the reduced FAD). The substrate, however, was subjected to the catalytic action of Y363. As the Y363 abstracted protons from the substrate, we observed a slow (14 μM mg^−1^ h^−1^) but steady increase in the 288 *m*/*z* signal, corresponding to d^1^-AD (Figure S16A). Based on the reaction mechanism, we assume that the deuteration predominantly proceeded at the 2β position yielding 2β-d^1^-AD. Moreover, after some initial delay, we also observed a significantly slower (3 μM mg^−1^ h^−1^ for 0.1 mM) rise in the 289 *m*/*z* signal (Figure S16B), which can be associated with 2α,2β-d^2^-AD. As a result, a 50–500-times slower isotope exchange of the second H atom for the deuteron was observed when compared to the exchange at the 2β position. The intensities of both signals, i.e., 288 and 289 *m*/*z* of d^1^-AD and d2-AD, were determined after corrections for the ^13^C isotopic [M + 1 + H]^+^ AD and d^1^-AD signals.

### 2.2. Transhydrogenation

As was demonstrated over 30 years ago by Itagaki [10], KstDs can catalyze transhydrogenation between 3-ketosteroids and 1-dehydro-3-ketosteroids, which act as both electrons and hydride and proton acceptors. Here, we conducted two types of experiments: (i) a transfer of deuteron labels introduced at either C1 or C2 position to the 1-dehydro-3-ketosteroids in H_2_O, or (ii) a transfer of protium labels between 3-ketosteroids and 1-dehydro-3-ketosteroids in D_2_O, thus observing the extent of the isotope exchange at Y363 or FAD. We tested the transhydrogenation process in the pH range of 6.5–8.2 with an equimolar ratio of ADD and 17-methyltestosterone (MT). The prepared reaction proceeded until an equilibrium was reached, regardless of the pH, with all four steroids (AD, ADD, MT and ∆^1^-MT) accounting for approximately 25% of the total steroid concentration (Supplementary Table S3 and Figure S17). Based on the previous studies of Itagaki et al., we expected to identify the pH optimum for transhydrogenation in between the pH optima for hydrogenation with BV (4.3) and dehydrogenation with DCPIP (6.5), i.e., a pH of around 5. However, in our experiments, we were limited by the AcmB stability, which started to decrease rapidly below a pH of 6.5. Therefore, we tested the pH dependence of transhydrogenation in the pH range of 6.5–8.0. Our experiments confirmed the steady increase in the specific activity of transhydrogenation when we moved from alkaline conditions of 8.0 (6.8 ± 0.5 µM h^−1^ mg^−1^) toward the expected pH optimum (the specific activity at pH 6.5 reached the value of 8.2 ± 0.5 µM h^−1^ mg^−1^—see Table S3). As a result, we conductedfurther transhydrogenation experiments at the best conditions of pH 6.5, where the rate was least limited by the slower hydrogenation, and the enzyme was still stable.

**Figure 4:**
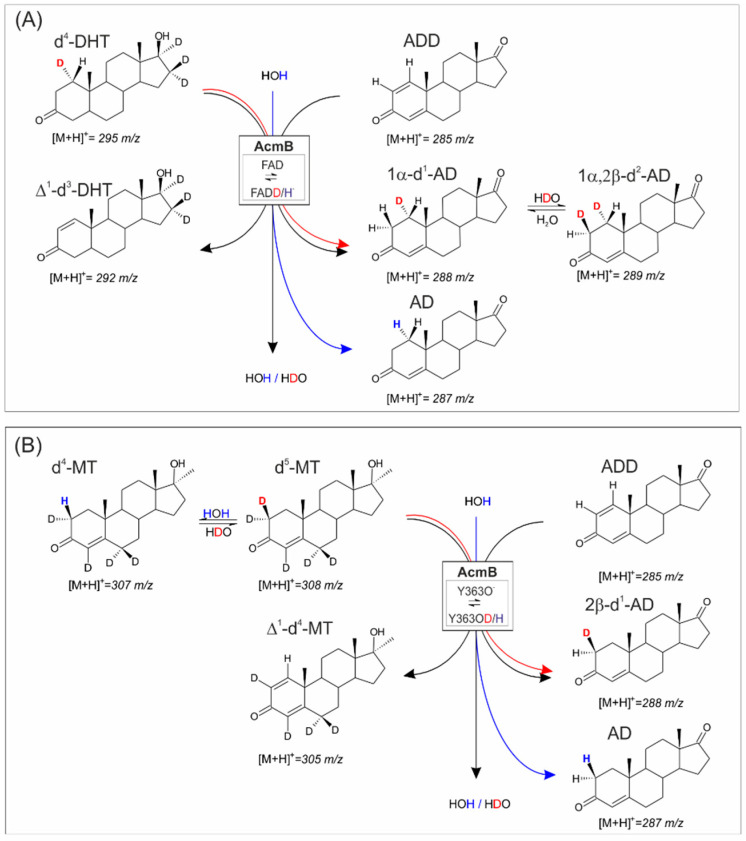
Scheme of transhydrogenation catalyzed by AcmB between ADD and deuterium-labelled substrates: (**A**) d^4^-dihydrotestosterone (d^4^-DHT), substituted at the 1α position, (**B**) d^5^-17-methyltestosterone (d^5^-MT), substituted at 2β and 2α. Red arrows depict the transhydrogenation of deuterium labels, while blue protium was derived from the solvent.

In the experiment with ADD and d^4^-DHT, we observed the predominant formation of 1α-d^1^-AD and Δ^1^-d^3^-DHT. The transfer of the 1α deuterium from d^4^-DHT ([M + H]^+^ = 295 *m*/*z*) to ADD ([M + H]^+^ = 285 *m*/*z*) resulted in the formation of 1α-d^1^-AD ([M + H]^+^ = 288 *m*/*z*) and ∆^1^-d^3^-DHT ([M + H]^+^ = 292 *m*/*z*) (Figure 4A). Although initially, the majority (51%) of the AD product contained transferred deuterons, from the very beginning of the reaction, we also observed the formation of the non-deuterated AD. This indicates an efficient isotope exchange of FADD^−^ with H_2_O (Figure 4A). As the reaction approached the equilibrium, even more deuterium was transferred to the solvent through isotope exchange at FAD. After 2 h, 60% of the AD was non-deuterated, and 21% of DHT was in the d^3^-DHT ([M + H]^+^ = 294 *m*/*z*). After 24 h, the majority (84%) of the observed AD contained only protium ([M + H]^+^ = 287 *m*/*z*) (Figure S18 and Table S4). The notably faster leaching of the deuteron labels from AD compared to DHT could be a result of the confirmed higher enzyme affinity for AD in comparison to DHT, which resulted in AD being more active in the transhydrogenation [14].

Another interesting effect that could be observed in this experiment was the detection of the heavier 3-ketosteroids than could be expected based on the initial mass of the standard and the transhydrogenation process. It should be first mentioned that the d^4^-DHT standard had a small but significant impurity of d^5^-DHT (9.9%), indicated by the elevated value of the 296 *m*/*z* signal (31% of the 295 *m*/*z* [M + H]^+^ signal instead of 21%). With the progress of the reaction, we observed an increase in the relative abundance of the 295 *m*/*z* signal with respect to 294 *m*/*z*, which suggests that the additional deuteration was resistant to the transhydrogenation process. As the absolute quantity of d^5^-DHT remained roughly the same during the whole process, we concluded that the label was not localized at the C1 or C2 atom. This conclusion was further corroborated by the analysis of the ∆^1^-d^3^-DHT isotopic pattern, which also exhibited an elevation of approx. 10% in the 294 *m*/*z* signal with respect to the [M + 1]^+^ signal of ∆^1^-d^3^-DHT.

Meanwhile, we observed a rather different effect in the case of AD formation. In addition to the already analyzed signals of AD ([M + H]^+^ = 287 *m*/*z*) and d^1^-AD ([M + H]^+^ = 288 *m*/*z*), we also observed a rise in the signal of 289 *m*/*z*, which oscillated between 6 and 8% of the total quantity of AD. Although we were unable to isolate and unequivocally identify the position of this additional deuteron, we could conclude that the deuteron was introduced at the 2β position in the process of the reverse exchange of H for D at Y363. Such an exchange must have been made possible by the significant leakage of deuterons from the C1 position in d^4^-DHT during transhydrogenation, which was observed from the formation of non-labelled AD. Inevitably, the exchange increased the concertation of D^+^ at the active site, which was observed from the introduction of an additional label to the AD product. Therefore, we postulate that the 289 *m*/*z* signal could be associated with 1α,2β-d^2^-AD.

In the experiment with ADD and d^5^-MT, we observed the formation of 2β-d^1^-AD and ∆^1^-d^4^-MT. The transfer of the 2β deuterium from d^5^-MT ([M + H]^+^ = 308 *m*/*z*) to ADD ([M + H]^+^ = 285 *m*/*z*) resulted in the formation of a mixture of AD ([M + H]^+^ = 287 *m*/*z*) and 2β-d^1^-AD ([M + H]^+^ = 288 *m*/*z*), as well as ∆^1^-d^4^-MT (M + H]^+^ = 305 *m*/*z*) (Figure 4B). In the case of the AD product, from the very beginning, we observed the predominant formation of the non-deuterated AD product (96–98% of [M + H]^+^ = 287 *m*/*z*), instead of 2β-d^1^-AD ([M + H]^+^ = 288 *m*/*z*) (Table S5), indicating a very efficient isotope D/H exchange at Y363OD with H_2_O.

Although from the start, the d^5^-MT was slightly contaminated with d^4^-MT (4% [M + H]^+^ = 307 *m*/*z*), during the reaction, we observed a rapid accumulation of d^4^-MT. Such a product could be formed in a non-productive process of dehydrogenation, when d^5^-MT binds to the reduced enzyme and undertakes deprotonation but is again deprotonated by protium instead of deuterium (as demonstrated in the hydrogenation experiment) or as a result of the transhydrogenation reaction between AD and ∆^1^-d^4^-MT. In the latter case, the transhydrogenation of protium resulted in the formation of d^4^-MT. After 6 h of the reaction, practically all the MT was in the d^4^-form. Above all, the experiment confirmed that the deuteron could be transmitted (albeit only as a trace label) from the C2 position in a 3-ketosteroid to a ∆^1^-ketosteroid (Figure 4B, Table S4).

**Figure 5:**
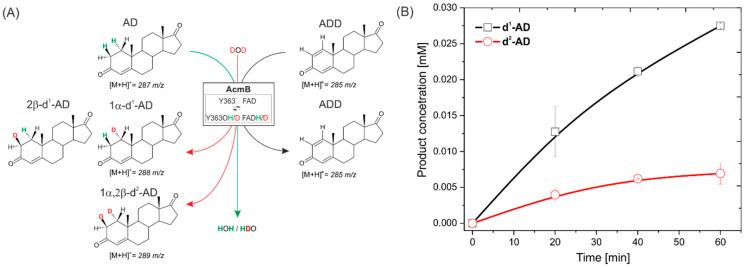
(**A**) Scheme of transhydrogenation catalyzed by AcmB between AD and ADD in D_2_O: (**B**) progress curve of the formation of deuterium-labelled AD.

Finally, to confirm the transfer of hydrogen from the solvent to the 1,2-hydrogenated steroid and evaluate the exchange rate, we conducted a transhydrogenation experiment between the equimolar AD and ADD in D_2_O. We expected to observe the formation of 1α or 2β-d^1^-AD and, to a minor extent, 1α,2β-d^2^-AD as a predominant product. Such expectations were based on the previous experiment, which indicated a high level of H/D exchange at both Y363 and FAD (Figure 5A). In order to avoid complicating the experiment with the subsequent reaction of the partially deuterated products with the ADD, we followed the reaction for only 60 min, during which less than 10% of the ADD was reduced and labelled with deuterium.

In the experiment, DAD detection was used to control the ADD and AD absolute concertation, while MS detection was used for the determination of the relative abundance of ions. As expected, over time, the emergence of the signals corresponding to d^1^-AD ([M + H]^+^ = 288 *m*/*z*) and d^2^-AD ([M + H]^+^ = 289 *m*/*z*) were observed (Figure 5B, Table S6 and Figure S3). Both signals co-occurred without any lag time for 289 *m*/*z*. This indicates that the formation of d^2^-AD, although less probable than the formation of d^1^-AD, occurred from the beginning of the reaction and was not a result of the transhydrogenation of d^1^-AD with ADD. Based on the MS, it was impossible to determine the relative ratio of 1α to 2β-d^1^-AD. However, the previous transhydrogenation tests and the observed higher rate of H/D exchange at Y363 suggest that the d^1^-AD could be predominantly labelled at the 2β position. The initial average rate of the ADD labelling with deuterons estimated for the first 40 min of the reaction was 41 μM mg^−1^ h^−1^ and 12 μM mg^−1^ h^−1^ for the introduction of one or two deuterons, respectively. This result is consistent with the slower rate of H/D exchange, which proceeds only at the C2 position when the enzyme is reduced.

A similar but not identical effect was previously described for KstD from *N. opaca* IMET 7030 [23] and *N. corallina* [10]. There, the transhydrogenation in D_2_O was conducted between progesterone and ADD in the pD range of 7.4–9.0. The authors observed the formation of the d^1^-AD product (with M^+^ = 287 *m*/*z*). Furthermore, the NMR analyses of the alkaline equilibration of deuterium-incorporated steroid confirmed the C2 position of the deuterium [10]. It seems that for the transhydrogenation process conducted at the alkaline pH, the H/D exchange at FAD was limited, unlike the reaction catalyzed by AcmB. At this point, we can only speculate what the cause of this effect was. However, the abovementioned H/D exchange, which leads to deuteration at the 1α-C1 position, is conducted by a reduced flavine cofactor, which may be in the negatively charged FADH^−^ or neutral form, FADH_2_. Although we do not know the pK_a_ of the reduced FAD in AcmB or KstD from *N. opaca* IMET 7030 and *N. corallina*, we can assume that the deprotonation of the FADH_2_ at higher pH values decreases the H/D exchange, resulting in the formation of d^1^-steroid. However, further transhydrogenation tests at higher pH values and the establishment of the pK_a_ of the reduced FAD are needed to test this hypothesis.

## 3. Material and Methods

### 3.1. Materials

All chemicals were purchased from Sigma-Aldrich (Germany), Tokyo Chemical Industry (Japan), BioShop (Canada), Carl Roth (Germany), or Chempur (Poland) unless otherwise specified. 4-Androsten-17α-methyl-17β-ol-3-one-2,2,4,6,6-d5 (d^5^-MT) was purchased from CDN Isotopes (Germany), 17β-hydroxy-5α-androstan-3-one-1,16,16,17-d4 (d^4^-DHT) was from Alsachim (France), and D_2_O was from Sigma-Aldrich (Germany).

### 3.2. Protein Expression and Purification

A gene encoding AcmB was cloned in a pMCSG7 vector, as described previously by Sofinska et al. [18], and transformed into calcium chloride chemically competent *E. coli* BL21(DE3) Magic. The AcmB expression and purification processes were described in our previous work [11]. The final enzyme concentration was determined spectrophotometrically at 280 nm (ε280 = 99,240 M^−1^ cm^−1^, ProtParam, ExPASy) and at 450 nm using a free FAD extinction coefficient in the case of AcmB (ε450 = 11,300 M^−1^ cm^−1^). For the 1,2-hydrogenation reaction, the frozen enzyme was lyophilized for 2–5 h and dissolved in D_2_O (99.9%) under anaerobic conditions (97% N_2_, 3% H_2_).

### 3.3. Spectrophotometric Assays

Spectrophotometric 1,2-hydrogenation assays were conducted in an anaerobic atmosphere (3/97 H_2_/N_2_ *v*/*v*) using 1.5 mM benzyl viologen, 0.15 mM Na-dithionite, 3.1 µM lyophilized AcmB and 0.4 mM ADD in 50 mM K_2_HPO_4_/KH_2_PO_4_ buffer with a pH of 6.5 in a 1 mL cuvette at 25 °C. The reactions were initiated by the addition of 8 μL of 50 mM substrate stock solution dissolved in 2-methoxyethanol (EGME). The change in the BV concentration was followed for 10 s at 575 nm (ε575 = 8900 M^−1^ cm^−1^) [19] using Shimadzu UV-1280. The pH optimum was established using 50 mM of citrate buffer for a pH range of 3.0–5.4 and 50 mM KH_2_PO_4_/K_2_HPO_4_ for a pH range of 6.5–8.0. 1,2-Dehydrogenation was measured analogically with 0.2 mM DCPIP and 0.2–0.4 mM AD.

The pH dependence of the maximum activity was modelled using Equation (1), which is applicable for a bell-shaped activity profile, where catalysis is deactivated by the deprotonation of an essential acid (*pK_a_*_1_) and protonation of an essential base (*pK_a_*_2_) [20]:

(1) $$V_{max}\left(pH\right)=\frac{V_{opt}}{1+10^{\left(pH-pK_{a1}\right)}+10^{\left(pK_{a2}-pH\right)}}=\;\;\;\frac{V_{opt}}{1+\frac{\left[H^{+}\right]}{K_{a1}}+\frac{K_{a2}}{\left[H^{+}\right]}}\;\;\;\;\;$$

### 3.4. 1,2-Hydrogenation

1,2-Hydrogenation on the 30 mL scale was prepared with D_2_O as the reaction medium (D_2_O, Sigma-Aldrich). A total of 17 mg of ADD in 1 mL of EGME, 5 mM benzyl viologen and 50 mM Na-dithionite were dissolved in 50 mM of phosphate buffer with a pH of 6.5 (estimated pD 6.9) [26], to which 9.8 µM (0.72 mg) of lyophilized AcmB was added to initiate the reaction. The reaction was conducted in a glass bottle with magnetic stirring under anaerobic conditions at room temperature for 24–48 h. An additional 5 mM of BV^2+^ and 25 mM Na-dithionite portions in D_2_O were added as needed, i.e., no purple colour of BV^0^ or a lack of reaction progress was observed. After 24 h, the additional portion of 7.4 µM (0.46 mg) of a catalyst was added as well. The reaction progress was monitored by HPLC and LC-MS.

For the comparison of 1,2-hydrogenation with 1,2-dehydrogenation, 1 mL batch reactors with 12.3 µM of fresh AcmB or 24.6 µM of AcmB after lyophilization were prepared. The reactions were conducted in 50 mM of K_2_HPO_4_/KH_2_PO_4_ buffer with a pH of 6.5 using 5.5 mM of DCPIP and 1.6 mM of AD. Samples were taken after 0, 1 and 2 h and analyzed by HPLC.

### 3.5. Transhydrogenation

The transhydrogenation experiments were performed in a volume of 1 mL under anaerobic conditions at 30 °C in a thermoblock using 1.2 µM of AcmB, 50 mM of phosphate buffer and a reaction mixture with 0.4 mM ADD and 0.4 mM MT (stocks prepared in 2-methoxyethanol) in the experiment with different pH values (6.5, 7.0 or 8.2). The reaction progress was monitored for 24 h by HPLC. The experiments on isotope labelling were prepared in a similar manner using 50 mM of phosphate buffer with a pH of 6.5, 1.2 µM of AcmB, 0.4 mM of ADD and 0.2 mM of d^4^-DHT or 0.4 mM of d^5^-MT. Samples were taken at 0, 5, 10, 30 and 120 min as well as after 24 h, or at 0, 5, 15, 30, 180 and 360 min as well as after 24 h in the experiments with d^4^-DHT or d^5^-MT, respectively, and analyzed by LC/MS.

The experiment in D_2_O was prepared analogically with 0.7 mM of AD and ADD, and the estimated pD was 6.9. Samples were taken at 0, 20, 40 and 60 min and analyzed by HPLC and LC/MS.

### 3.6. HPLC and LC-MS Detection

The 10 µL samples from the reaction were dissolved in 190 µL acetonitrile and analyzed with HPLC-DAD using Agilent 1100 VL LC/DAD/MSD. The separation was conducted using a HALO 90 Å RP-Amide column (2.7 μm, 2.1 × 75 mm, Advanced Materials Technology) at 30 °C in an isocratic mode using 35%/65% ACN/H_2_O mobile phase at a flow rate of 0.4 mL/min.

The LC-MS analyses were conducted using the Agilent Technologies LC System 1260 equipped with the Agilent Technologies 6410 Triple Quad LC/MS System (gas temperature: 330 °C, gas flow: 11 L/min, nebulizer: 15 psi, capillary: 4000 V), using an ESI ion source in a positive ion scan mode with a 275–310 *m*/*z* mass range and fragmentor at 135 V. The LC separation conditions were the same as in the HPLC-DAD experiments. 

### 3.7. Product Purification and NMR Analysis

AD obtained in 30 mL was purified either by the SPE method using StrataX columns and 500 mg (Phenomenex) of isopropanol as an elution solvent or by liquid–liquid extraction with ethyl acetate. Both methods were followed by extraction with CHCl_3_ (3 × 300 mL), drying (MgSO_4_) and concentration in vacuo. The products were separated using preparative TLC plates (Silica Gel GF, 20 × 20 cm, 500 μm, Analtech) and a hexane/acetone mixture (3:1, *v*/*v*) as an eluent. The structure of the reaction products was determined using ^1^H NMR, ^13^C NMR and correlation spectroscopy. The NMR spectra were recorded using a DRX 600 MHz Bruker spectrometer and measured in CDCl_3_ or DSMO-d_6_ (Supplementary Materials).

^1^H NMR (400 MHz) (ppm) (CDCl_3_) δ: 0,92 (s, 3H, 18-H); 0.99 (ddd, 1H, J = 12.3, 10.9, 4.2 Hz, 9-H); 1.12 (qd, 1H, J = 13.3, 4.4 Hz, 7-Hα); 1.21 (s, 3H, 19-H); 1.24–1.34 (m, 2H, 12-Hα, 14-H); 1.49 (qd, 1H, J = 13.1, 4.1 Hz, 11-Hβ); 1.57 (tt, 1H, J = 12.5, 9.2 Hz, 15-Hβ); 1.69 (ddd, 1H, J = 13.6, 6.9, 4.1 Hz, 11-Hα) 1.75 (td, 1H, J = 10.9, 3.5 Hz, 8-H); 1.89 (ddd, 1H, J = 12.9, 3.8, 2.9 Hz, 12-Hβ); 1.94–2.03 (m, 3H, 1-Hβ, 7-Hβ, 15-Hα); 2.11 (dt, 1H, J = 19.2, 9.1 Hz, 16-Hα); 2.30–2.36 (m, 2H, 2-Hα, 6-Hα); 2.42 (ddd, 1H, J = 13.6, 5.2, 1.7 Hz, 6-Hβ); 2.48 (dd, 1H, J = 19.2, 8.9 Hz, 16-Hβ); 5.75 (s, 1H, 4-H). ^13^C NMR (151 MHz) (ppm) (CDCl_3_) δ: 35.36 (t, J = 19.3Hz, C-1); 33.67 (t, J = 19.6Hz, C-2); 199.61 (C-3); 124.36 (C-4); 170.52 (C-5); 32.72 (C-6); 30.92 (C-7); 35.30 (C-8); 53.92 (C-9); 38.71 (C-10); 20.47 (C-11); 31.43 (C-12); 47.66 (C-13); 51.00 (C-14); 21.90 (C-15); 35.90 (C-16); 220.54 (C-17); 13.85 (C-18); 17.57 (C-19).

^1^H NMR (400 MHz) (ppm) (DSMO-d_6_) δ: 0,83 (s, 3H, 18-H); 0.94 (ddd, 1H, J = 12.3, 10.9, 4.1 Hz, 9-H); 1.12 (qd, 1H, J = 12.7, 4.2 Hz, 7-Hα); 1.17 (s, 3H, 19-H); 1.18 (td, 1H, J = 13.1, 4.3 Hz, 14-H); 1.26 (ddd, 1H, J = 12.7, 11.1, 5.9 Hz, 12-Hα) 1.49 (qd, 1H, J = 13.3, 4.1 Hz, 11-Hβ); 1.52 (tt, 1H, J = 12.3, 9.1 Hz, 15-Hβ); 1.59 (qd, 1H, J = 13.7, 3.8 Hz, 11-Hα) 1.67 (ddd, 1H, J = 12.7, 3.9, 2.9 Hz, 12-Hβ); 1.73 (qd, 1H, J = 11.1, 3.4 Hz, 8-H); 1.85–1.96 (m, 3H, 1-Hβ, 7-Hβ, 15-Hα); 2.01 (dt, 1H, J = 19.1, 9.0 Hz, 16-Hα); 2.12–2.17 (m, 1H, 2-Hα); 2.28 (ddd, 1H, J = 14.5, 4.0, 2.4 Hz, 6-Hα); 2.37–2.46 (m, 2H, 6-Hβ, 16-Hβ); 5.65 (s, 1H, 4-H). ^13^C NMR (151 MHz) (ppm) (DSMO-d6) δ: 34.62 (t, J = 18.8Hz, C-1); 33.20 (t, J = 19.7Hz, C-2); 198.12 (C-3); 123.30 (C-4); 170.73 (C-5); 31.82 (C-6); 30.46 (C-7); 34.40 (C-8); 53.10 (C-9); 38.20 (C-10); 19.91 (C-11); 31.05 (C-12); 46.91 (C-13); 49.99 (C-14); 21.35 (C-15); 35.30 (C-16); 219.53 (C-17); 13.36 (C-18); 16.94 (C-19).

QM:MM calculation

The model of the AcmB in complex with AD was based on the structure deposited in PDB (PDB code: 7P18). The details of the model preparation are described in detail in our recent report [9]. Briefly, the ADD was replaced with AD, making use of the Kabsch algorithm, and the protonation was determined by propKa3.1 [27,28] for a pH of 6.5, and the missing MM parameters were obtained using QM calculations in Gaussian 16 (https://gaussian.com/citation/) at the B3LYP/6-31G(d,p) level or the RESP ESP charge DataBase (R.E.DD.B) [29]. The total +1 charge of the model was neutralized by one Cl^−^ ion, and the model was soaked with TIP3P water molecules in a 94.4 × 78.8 × 78.4 Å3 box. After 60 ns of MD simulation in AMBER, the selected model was subjected to QM:MM modelling. The QM layer consisted of Y363, a fragment of FAD and a substrate, while the rest of the model was described with an AMBER forcefield, as implemented in the fDynamo library [30,31]. The reaction was modelled using the umbrella sampling technique [32]. For each step, a reaction coordinate was defined as an antisymmetric combination of selected distances. Then, a series of QM:MM MD simulations with an additional parabolic penalty potential (2500 kJ/Å^2^ mol) was carried out. One-dimensional energy scans generated the initial geometries for these calculations. Every window consisted of 5 ps of relaxation and 20 ps of production MD. The weighted histogram analysis method was used to produce the potential of mean force (PMF) [33]. Then, single-point calculations at the B3LYP/6-311++G(2d,2p) level of theory were applied to correct the obtained profiles with the energy spline function. Finally, the structures related to the stationary points were optimized at the B3LYP/6-31G(d,p) level of theory using the Baker algorithm [34] and micro–macro iteration scheme [35]. The energies of the stationary points were corrected using zero-point energy corrections calculated for deuterated and non-deuterated substrates.

## 4. Conclusions

3-Ketosteroid Δ^1^-dehydrogenase from *Sterolibacterium denitrificans* catalyzes the regio- and enantioselective 1,2-hydrogenation of Δ^1^-3-ketosteroids, introducing H atoms to the 1α and 2β positions in ring A of the steroid core. The AcmB pH optima for the hydrogenation and dehydrogenation processes are lower by approximately 3.5–4 units compared to KstD from *N. corallina*. At a pH of 6.5, the hydrogenation is over 250 slower than 1,2-dehydrogenation. If the 1,2-hydrogenation is conducted in D_2_O, the deuterons are introduced enantiospecifically, yielding a d^2^-substituted product. Significantly, the low pH hydrogenation catalyzed by AcmB leads to different products than high-pH hydrogenation catalyzed by KstD, as investigated by Igataki et al. Furthermore, the deuteration at the 2β positions reduces the enantioselectivity of C2-H activation, which results in the introduction of the third deuteron, most probably at the 2α position. A high degree of H/D exchange is observed during transhydrogenation at Y363, which activates the C2-H bond, and, to a smaller extent, also at the reduced FAD, which transfers the hydride to C1.

## Figures and Tables

**Table 1:** Results of the QM:MM modelling of 1,2-dehydrogenations. The ∆(E + ZPE) energies were provided in kcal/mol for 1α,2β-deuterated AD and non-labelled AD.

	∆(E + ZPE) (kcal/mol)			
	1α,2β-d^2^-AD		AD	
	2β Attack	2α Attack	2β Attack	2α Attack
E:S	0	0	0	0
TS1	10.85	14.19	9.94	14.05
E:I	3.13	3.13	2.92	2.92
TS2	13.39	13.50	12.59	12.59
E:P	−9.63	−9.46	−9.45	−9.45

## Data Availability

Not applicable.

## Supplementary Materials

The following supporting information can be downloaded at: https://www.mdpi.com/article/10.3390/ijms232314660/s1.
