# Supplementary material for: 1,2-Hydrogenation and Transhydrogenation Catalyzed by 3-Ketosteroid Δ1-Dehydrogenase from Sterolibacterium denitrificans—Kinetics, Isotope Labelling and QM:MM Modelling Studies
Source: Int J Mol Sci. 2022 Nov 24;23(23):14660. doi: 10.3390/ijms232314660 (PMC9736390; doi:10.3390/ijms232314660)
Supplement: Supplementary file 1 [file ijms-23-14660-s001.zip › SI.docx]

Supplementary materials

1,2-Hydrogenation and transhydrogenation catalyzed by 3-ketosteroid Δ^1^-dehydrogenase from *Sterolibacterium denitrificans* – kinetics, isotope labelling and QM: MM modelling studies

Agnieszka M. Wojtkiewicz^1^, Michał Glanowski^1^, Piotr Waligórski^2^, Tomasz Janeczko^3^, Maciej Szaleniec^1^

^1^Jerzy Haber Institute of Catalysis and Surface Chemistry, Polish Academy of Sciences, Niezapominajek 8, 30-239 Krakow, Poland

^2^The Franciszek Górski Institute of Plant Physiology, Polish Academy of Sciences, Niezapominajek 8, PL30239 Krakow, Poland

^3^Department of Food Chemistry and Biocatalysis, Wrocław University of Environmental and Life Sciences, Norwida 25, PL50375 Wrocław, Poland

Index

[1,2-Hydrogenation 2](#_Toc117797374)

[NMR analyses 4](#_Toc117797375)

[Transhydrogenation 13](#_Toc117797376)

## 1,2-Hydrogenation

| (A)   | |
| --- | --- |
| (B)   | ADD, RT=2.388 min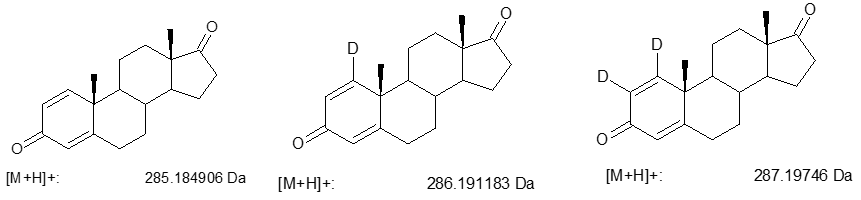 |
|  | AD, RT=3.569 min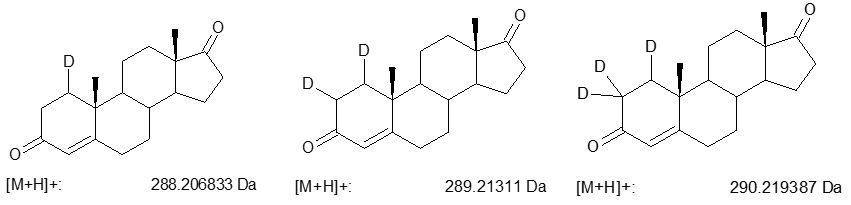 |

Figure S1. LC-MS analysis of 1,2-hydrogenation of ADD to AD catalysed by AcmB. Chromatogram with separated analytes (A). MS signals of a substrate and a product with corresponding structures that explains the observed m/z.

Table S1. The comparison of AcmB activity in the batch system depended on the direction of the reaction and the type of enzyme applied to the experiments.

| Reaction | Total volume [ml] | Enzyme type | Enzyme volume [ml] | Volume activity [%_product_ · h^-1^ · enzyme volume^-1^] | Relative activity [%] |
| --- | --- | --- | --- | --- | --- |
| AD → ADD | 1 | Fresh | 0.04 | 2.97 | 100 |
| AD → ADD | 1 | Lyophilized | 0.08 | 0.92 | 31 |
| ADD → AD | 30 | Lyophilized | 2.4 | 0.03 | 1 |

## NMR analyses

Figure S2. ^1^H NMR spectrum of 1α,2β-d^2^-androstenedione (CDCl_3_, 600 MHz)

Figure S3. ^13^C NMR spectrum of 1α,2β-d^2^-androstenedione (CDCl_3_, 151 MHz).

Figure S4. COSY spectrum of 1α,2β-d^2^-androstenedione (CDCl_3_, 600 MHz).

Figure S5. HSQC spectrum of 1α,2β-d^2^-androstenedione (CDCl_3_, 151 MHz).

Figure S6. HMBC spectrum of 1α,2β-d^2^-androstenedione (CDCl_3_, 151 MHz).

Figure S7. Part of the HMBC spectrum of 1α,2β-d^2^-androstenedione (CDCl_3_, 151 MHz)

Figure S8. ^1^H NMR spectrum of 1α,2β-d^2^-androstenedione (DMSO-*d*_6_, 600 MHz)

Figure S9. ^13^C NMR spectrum of 1α,2β-d^2^-androstenedione (DMSO-*d*_6_, 151 MHz)

Figure S10. COSY spectrum of 1α,2β-*d*_2_-androstenedione (DMSO-*d*_6_, 600 MHz)

Figure S11. HSQC spectrum of 1α,2β-*d*_2_-androstenedione (DMSO-*d*_6_, 151 MHz)

Figure S12. HMBC spectrum of 1α,2β-*d*_2_-androstenedione (DMSO-*d*_6_, 151 MHz)

Figure S13. Part of the HMBC spectrum of 1α,2β-*d*_2_-androstenedione (DMSO-*d*_6_, 151 MHz)

Figure S14. ^1^H NMR spectrum of androstenedione (DMSO-*d*_6_, 600 MHz)

1β

1β

Figure S15a. Part of the ^1^H NMR spectrum of androstenedione (brown line)
and 1α,2β-*d*_2_-androstenedione (green line) (DMSO-*d*_6_, 600 MHz)

11α

1α and 11α

Figure S15b. Part of the ^1^H NMR spectrum of androstenedione (**1**) (brown line)
and 1α,2β-*d*_2_-androstenedione (green line) (DMSO-*d*_6_, 600 MHz)

2α

2α

Figure S15c. Part of the ^1^H NMR spectrum of androstenedione (**1**) (brown line)
and 1α,2β-*d*_2_-androstenedione (green line) (DMSO-*d*_6_, 600 MHz)

Table S2. MS Analysis of 1,2-hydrogenation of ADD catalyzed by AcmB. In the table were presented MS signals of the quasi molecular ion [M+H]^+^ and its [M+1+H]^+^ or [M+2H]^+^. The theoretical isotopic pattern of ADD quasi molecular ion (C_19_H_24_O_2_+H) and d^2^-AD quasi molecular ion (C_19_H_24_D_2_O_2_+H) are 100, 21, 2.5 % of 285 m/z and 290 m/z, respectively.

|  | ADD | | AD | |
| --- | --- | --- | --- | --- |
| t [min] | m/z | % of signal | m/z | % of signal |
| 0 | 285 | 100 | 289 | 0 |
|  | 286 | 21 | 290 | 0 |
|  | 287 | 3 | 291 | 0 |
| 1 | 285 | 100 | 289 | 100 |
|  | 286 | 21 | 290 | 30 |
|  | 287 | 3 | 291 | 20 |
| 2.5 | 285 | 100 | 289 | 100 |
|  | 286 | 21 | 290 | 25 |
|  | 287 | 3 | 291 | 10 |
| 3.5 | 285 | 100 | 289 | 100 |
|  | 286 | 38 | 290 | 22 |
|  | 287 | 5 | 291 | 7 |
| 5 | 285 | 100 | 289 | 100 |
|  | 286 | 21 | 290 | 22 |
|  | 287 | 3 | 291 | 5 |
| 21 | 285 | 100 | 289 | 100 |
|  | 286 | 21 | 290 | 21 |
|  | 287 | 3 | 291 | 4 |
| 23 | 285 | 100 | 289 | 100 |
|  | 286 | 22 | 290 | 22 |
|  | 287 | 3 | 291 | 3 |
| 24 | 285 | 100 | 289 | 100 |
|  | 286 | 10 | 290 | 21 |
|  | 287 | 3 | 291 | 3 |
| 25 | 285 | 100 | 289 | 100 |
|  | 286 | 21 | 290 | 21 |
|  | 287 | 3 | 291 | 3 |
| 45 | 285 | 100 | 289 | 100 |
|  | 286 | 23 | 290 | 21 |
|  | 287 | 6 | 291 | 3 |
| after extraction | 285 | 100 | 289 | 100 |
|  | 286 | 42 | 290 | 38 |
|  | 287 | 2 | 291 | 8 |

A. B.

Figure S16. Formation of 2β-d^1^-AD ([M+H]+ = 288 m/z) and 2α,2β-d^2^-AD ([M+H]^+^ = 289 m/z) from AD ([M+H]^+^ = 287 m/z) in D_2_O catalysed by the AcmB reduced with BV^+^.

## Transhydrogenation

Table S3. Transhydrogenation of 1,4-androstdien-3,17-dione (ADD) and 17- methyltestosterone (MT) by AcmB in different pHs. Reaction rates of each product formation were determined from HPLC for the first 15 min of the reaction.

|  | AD [mM] | | | Δ^1^-MT [mM] | | | Reaction rate [µM h^-1^ mg^-1^] | | |
| --- | --- | --- | --- | --- | --- | --- | --- | --- | --- |
|  | 0 h | 24 h | 0 h | | 24 h | AD | | Δ^1^-MT |  |
| pH=6.5 | 0.02 ± 0.00 | 0.20 ± 0.02 | 0.02 ± 0.00 | | 0.20 ± 0.02 | 8.2 ± 0.5 | | 8.1 ± 0.5 |  |
| pH=7.0 | 0.02 ± 0.00 | 0.18 ± 0.02 | 0.01 ± 0.00 | | 0.18 ± 0.02 | 7.1 ± 0.4 | | 7.1 ± 0.4 |  |
| pH=8.2 | 0.02 ± 0.00 | 0.19 ± 0.01 | 0.01 ± 0.00 | | 0.19 ± 0.01 | 6.8 ± 0.5 | | 6.8 ± 0.5 |  |

A. B.

Figure S17. Progress of transhydrogenation between ADD and MT at different pHs, where A) conversion to AD [mM] and B) to Δ^1^-MT [mM].

Figure S18. LC-MS analysis of transhydrogenation between ADD and d^4^-DHT catalyzed by AcmB. (A). Chromatogram with separated analytes (B) MS signals of substrates and products.

Table S4. MS analysis of transhydrogenation between ADD and d^4^-DHT catalyzed by AcmB. In the table were presented MS signals of [M+H]^+^ and their isotope signals [M+1+H]^+^ or [M+2+H]^+^. The theoretical isotopic pattern of ADD quasi molecular ion (C_19_H_24_O_2_+H) and d^2^-AD quasi molecular ion (C_19_H_24_D_2_O_2_+H) are 100, 21, 2.5% of 285 m/z and 290 m/z, respectively.

| Time | ADD | | DHT | | AD | | Δ^1^-DHT | |
| --- | --- | --- | --- | --- | --- | --- | --- | --- |
| [min] | m/z | % of signal | m/z | % of signal | m/z | % of signal | m/z | % of signal |
| 0.00 | 285 | 100% | 294 | 11% | 287 | 0% | 292 | 0% |
|  | 286 | 21% | 295 | 100% | 288 | 0% | 293 | 0% |
|  | 287 | 3% | 296 | 32% | 289 | 0% | 294 | 0% |
|  |  |  | 297 | 10% |  |  |  |  |
| 5.00 | 285 | 100% | 294 | 14% | 287 | 61% | 292 | 100% |
|  | 286 | 21% | 295 | 100% | 288 | 100% | 293 | 68% |
|  | 287 | 3% | 296 | 38% | 289 | 42% | 294 | 33% |
|  |  |  | 297 | 14% |  |  |  |  |
| 10.00 | 285 | 100% | 294 | 18% | 287 | 46% | 292 | 100% |
|  | 286 | 22% | 295 | 100% | 288 | 100% | 293 | 60% |
|  | 287 | 3% | 296 | 37% | 289 | 29% | 294 | 25% |
|  |  |  | 297 | 16% |  |  |  |  |
| 30.00 | 285 | 100% | 294 | 17% | 287 | 60% | 292 | 100% |
|  | 286 | 21% | 295 | 100% | 288 | 100% | 293 | 56% |
|  | 287 | 3% | 296 | 39% | 289 | 35% | 294 | 23% |
|  |  |  | 297 | 14% |  |  |  |  |
| 120.00 | 285 | 100% | 294 | 25% | 287 | 133% | 292 | 100% |
|  | 286 | 20% | 295 | 100% | 288 | 100% | 293 | 71% |
|  | 287 | 3% | 296 | 39% | 289 | 36% | 294 | 39% |
|  |  |  | 297 | 19% |  |  |  |  |
| 1440.00 | 285 | 100% | 294 | 31% | 287 | 309% | 292 | 100% |
|  | 286 | 21% | 295 | 100% | 288 | 100% | 293 | 33% |
|  | 287 | 3% | 296 | 35% | 289 | 39% | 294 | 23% |
|  |  |  | 297 | 15% |  |  |  |  |

Table S5. MS Analysis of transhydrogenation with ADD and d^5^-MT as co-substrates catalysed by AcmB. In the table were presented MS signals of [M+H]^+^ and their isotope signals [M+1+H]^+^ or [M+2+H]^+^. The theoretical isotopic pattern of ADD and AD quasi molecular ions (C_19_H_24_O_2_+H) and (C_19_H_26_O_2_+H) are 100, 21, 2.5% of 285 m/z and 287 m/z, respectively; d^5^-MT and Δ^1^-d^4^-MT quasi molecular ions (C_20_H_25_D_2_O_2_+H) and (C_20_H_24_D_2_O_2_+H) are 100, 22, 2.7% of 308 m/z and 305 m/z, respectively.

| Time | ADD | | d^5^-MT | | AD | | Δ^1^-d^4^-MT | |
| --- | --- | --- | --- | --- | --- | --- | --- | --- |
| [min] | m/z | % of signal | m/z | % of signal | m/z | % of signal | m/z | % of signal |
| 0.00 | 285 | 100% | 307 | 100.00% | 287 | 0% | 305 | 100% |
|  | 286 | 21% | 308 | 288072.93% | 288 | 0% | 306 | 31% |
|  | 287 | 3% | 309 | 49052.39% | 289 | 0% | 307 | 10% |
| 5.00 | 285 | 100% | 307 | 100.00% | 287 | 100% | 305 | 100% |
|  | 286 | 20% | 308 | 621.01% | 288 | 25% | 306 | 23% |
|  | 287 | 4% | 309 | 133.66% | 289 | 9% | 307 | 4% |
| 15.00 | 285 | 100% | 307 | 100.00% | 287 | 100% | 305 | 100% |
|  | 286 | 22% | 308 | 155.60% | 288 | 24% | 306 | 22% |
|  | 287 | 4% | 309 | 33.78% | 289 | 6% | 307 | 3% |
| 30.00 | 285 | 100% | 307 | 100.00% | 287 | 100% | 305 | 100% |
|  | 286 | 22% | 308 | 71.52% | 288 | 24% | 306 | 22% |
|  | 287 | 5% | 309 | 15.05% | 289 | 9% | 307 | 4% |
| 180.00 | 285 | 100% | 307 | 100.00% | 287 | 100% | 305 | 100% |
|  | 286 | 22% | 308 | 42.12% | 288 | 23% | 306 | 22% |
|  | 287 | 4% | 309 | 7.62% | 289 | 4% | 307 | 3% |
| 360.00 | 285 | 100% | 307 | 100.00% | 287 | 100% | 305 | 100% |
|  | 286 | 23% | 308 | 42.59% | 288 | 23% | 306 | 22% |
|  | 287 | 4% | 309 | 8.05% | 289 | 4% | 307 | 3% |
| 1440.00 | 285 | 100% | 307 | 100.00% | 287 | 100% | 305 | 100% |
|  | 286 | 21% | 308 | 41.34% | 288 | 22% | 306 | 23% |
|  | 287 | 5% | 309 | 7.99% | 289 | 4% | 307 | 3% |

Table S6. MS Analysis of transhydrogenation with ADD and AD as co-substrates catalysed by AcmB. In the table were presented MS signals of [M+H]^+^ and their isotope signals [M+1+H]^+^ or [M+2+H]^+^. The theoretical isotopic pattern of ADD and AD quasi molecular ions (C_19_H_24_O_2_+H) and (C_19_H_26_O_2_+H) are 100, 21, 2.5% of 285 m/z and 287 m/z, respectively;

|  | ADD | | AD | | Increase of signal that corresponds to the addition of deuterium in AD |
| --- | --- | --- | --- | --- | --- |
| t [min] | m/z | % of signal | m/z | % of signal |  |
| 0 | 285 | 100% | 287 | 100% | - |
|  | 286 | 21% | 288 | 23% | 1% |
|  | 287 | 3% | 289 | 3% | 1% |
| 20 | 285 | 100% | 287 | 100% | - |
|  | 286 | 21% | 288 | 24% | 3% |
|  | 287 | 3% | 289 | 4% | 1% |
| 40 | 285 | 100% | 287 | 100% | - |
|  | 286 | 21% | 288 | 25% | 4% |
|  | 287 | 3% | 289 | 5% | 2% |
| 60 | 285 | 100% | 287 | 100% | - |
|  | 286 | 21% | 288 | 26% | 5% |
|  | 287 | 3% | 289 | 5% | 2% |

.
